# Conserved role of fructokinase-like protein 1 in chloroplast development revealed by a seedling-lethal albino mutant of pepper

**DOI:** 10.1093/hr/uhab084

**Published:** 2022-01-20

**Authors:** Chunmei Shi, Xinyan Shen, Zhiying Zhang, Yuhong Zhou, Rong Chen, Jingying Luo, Yaping Tang, Yongen Lu, Feng Li, Bo Ouyang

**Affiliations:** Key Laboratory of Horticultural Plant Biology (Ministry of Education), Huazhong Agricultural University, Wuhan 430070, China

Dear Editor,

Chloroplasts perform photosynthesis and thus drive the growth, development, and reproduction of plants. Mutations in genes that encode chloroplast proteins frequently lead to chlorophyll deficiencies. Studies on chlorophyll-deficient mutants have led to major advances in our knowledge of chloroplast- and photosynthesis-associated genes in model plants. Similarly, studies on chlorophyll-deficient mutants will contribute to our understanding of chloroplast development and function in solanaceous crops, such as pepper, thus helping us to rationally manipulate photosynthesis for crop improvement. Pepper is a globally important vegetable that is used as a spice, food, and medicine.

Chloroplast genes are transcribed by either the plastid-encoded or nuclear-encoded RNA polymerase (PEP or NEP) [1]. PEP is a multisubunit enzyme that consists of core subunits encoded by the plastid-localized genes *rpoA*, *rpoB*, *rpoC1*, and *rpoC2*. PEP-transcribed genes are essential for efficient photosynthesis and normal growth [2]. Loss-of-function mutants deficient in PEP-associated proteins (PAPs) develop albino or pale-green leaves [3, 4]. Thioredoxin Z (TRXz) and a fructokinase-like protein (FLN) associate with the PEP complex and contribute to the redox regulation of PEP [5]. Deficiencies in either TRXz or FLN1 inhibit both PEP and photoautotrophic growth [6]. In *Arabidopsis*, TRXz and FLN1 contribute to PEP-mediated transcription and chlorophyll accumulation [7]. In rice, OsTRXz and two PAPs, WLP2/OsFLN1 and HSA1/OsFLN2, form a TRX-FLN complex that regulates PEP-mediated transcription and consequently affects chloroplast development [8, 9]. Although PAPs have been studied in *Arabidopsis* and rice, few studies on PAPs have been performed with solanaceous crops such as pepper.

We report the positional cloning of a mutant allele responsible for an albino seedling-lethal phenotype in the miniature pepper cultivar MiniPep (*Capsicum annuum*). This mutant was unable to produce true leaves and eventually died at the seedling stage (Fig. 1a). Chlorophyll *a* and *b* were barely detectable in the 14-d-old seedlings of the mutant (*e1493*) relative to the wild type (Fig. 1b). Transmission electron microscopy demonstrated the abnormal shape and ultrastructure of the plastids in *e1493* relative to those in the wild type. The plastid in *e1493* is round or nearly round, and the thylakoid membranes were barely detectable (Fig. 1c). Genetic analysis indicated that a recessive nuclear gene was responsible for these phenotypes.

**Figure 1:**
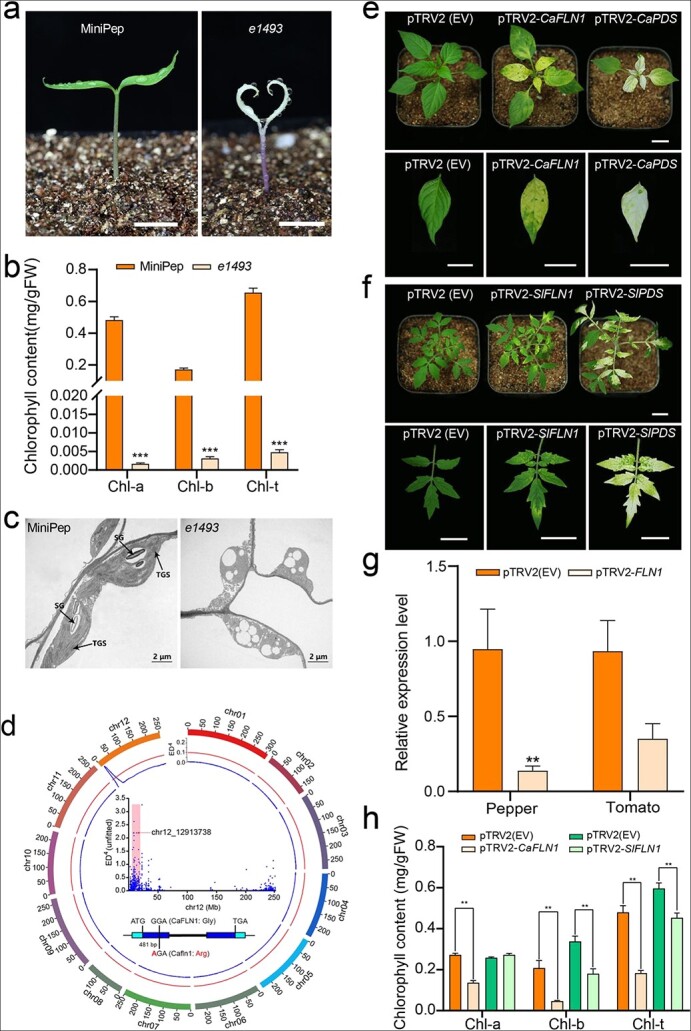
Conserved role of fructokinase-like protein 1 in chloroplast development revealed by the seedling-lethal albino mutant of pepper, *e1493*. (a) Phenotypes of 14-d-old wild-type (MiniPep) and mutant (*e1493*) seedlings. Scale bar, 1 cm. (b) Chlorophyll content of the cotyledons from MiniPep and *e1493*. Error bars indicate standard deviation. ^***^P < 0.000005, Student’s *t*-test. (c) Ultrastructure of chloroplasts from the cotyledons of MiniPep and *e1493* observed using transmission electron microscopy. SG, starch grain; TGS, thylakoid grana stack. (d) Mapping of a missense allele in *CaFLN1*. The mutant allele was mapped to chromosome 12 (Circos plot) and to an interval between 6.1 M and 18.9 M using bulked segregant RNA-seq (BSR). The causative SNP (chr12_12913738) is presented inside the circle. The horizontal thick line represents an intron. The light and navy blue boxes represent untranslated regions (UTRs) and exons, respectively. The translational start codon (ATG), translational stop codon (TGA), and the G-to-A missense mutation are indicated with vertical lines. The missense mutation is located at 481 bp relative to the first bp of the translational start codon and changes a glycine (Gly) to an arginine (Arg) residue in the CaFLN1 protein. (e) VIGS of *CaFLN1* in pepper. (f) VIGS of *SlFLN1* in tomato. Plants inoculated with an *Agrobacterium* strain containing the pTRV2 empty vector (pTRV2 (EV)) were used as a negative control. For a positive control, plants were inoculated with an *Agrobacterium* strain containing the pTRV2-PDS vector, which contains a fragment of the phytoene desaturase (*PDS*) gene from either pepper (e) or tomato (f). Scale bar, 2 cm. (g) Relative expression of *FLN1* in pepper and tomato plants subjected to VIGS. Thirty-day-old plants were analyzed. Gene expression was normalized to the expression of *UBI-3* in pepper and *SlFRG37* in tomato. (h) Chlorophyll content in chlorotic leaves from pepper and tomato plants subjected to VIGS. Error bars indicate standard deviation (n = 3). ^*^P < 0.05, ^**^P < 0.01, Student’s *t*-test.

To clone the causal gene, we performed a bulked segregant RNA-seq (BSR) analysis with an F_2_ population prepared by crossing a plant that was heterozygous for the mutant allele from *e1493* with the PC69 cultivar of *C. annuum*. We extracted RNA from pools of albino and wild-type tissue and used equivalent amounts of tissue from 30 seedlings for each pool. The BSR analysis indicated that the causal gene was located between 6.1 Mb and 18.9 Mb on chromosome 12 in the CM334 genome (Fig. 1d, Fig. S1a). To identify the target gene, we re-sequenced the wild type (MiniPep) and *e1493*. We identified 49 candidate single-nucleotide polymorphisms (SNPs) in the 12.8-Mb interval (Table S1) that were homozygous for G/C to A/T substitutions [10]. After annotation, only one SNP (chr12_12913738) with a ΔSNP index of −0.86 (Table S2) was found to be a missense mutation and was located in *CA.PGAv.1.6.scaffold321.29*, which we named *CaFLN1*. We independently demonstrated the presence of this missense mutation in *CaFLN1* using Sanger sequencing (Fig. S1b). The combination of BSR, parental resequencing, and SNP filtering enabled us to rapidly identify the gene responsible for the albino phenotype in *e1493*. We expect that this strategy will be generally useful for cloning EMS alleles in crops. Sequence analysis showed that *CaFLN1* is homologous to *AtFLN1* (AT3G54090) from *Arabidopsis*, which encodes a fructokinase-like protein. The mutant of *AtFLN1* also shows an albino phenotype, and FLN1 interacts with the plastid-localized TRXz to promote chloroplast development [7]. *CaFLN1* also showed high similarity with *WLP2* (*Os01t0851000*), and *wlp2* mutants are also albino. Moreover, WLP2 interacts with OsTRXz to form a TRX-FLN complex that promotes chloroplast development [8]. Similar to the *cafln1* mutant, the *osfln1* (*wlp2*) mutant is also an albino seedling-lethal mutant in rice [9]. A bioinformatics analysis provides evidence that *CaFLN1* is targeted to the chloroplast and that *CaFLN1* is preferentially expressed in leaves (Fig. S1c–d).

*CaFLN1* contains two exons and one intron with a 1392-bp open reading frame that encodes a protein containing 464 amino acid residues*.* The mutation changes a G to an A at position 481 relative to the first bp of the first exon, changing a glycine (Gly) to an arginine (Arg) residue in the CaFLN1 protein (Fig. 1d). FLN1 contains the conserved fpkB domain (Fig. S2). Our phylogenetic analysis indicates that there are four fpkB subfamilies: fructokinases (RFKs), fructokinase-like protein (FLN), adenosine kinase (ADK), and other kinases. The FLNs and RFKs are located on the same branch (Fig. S3). An amino acid sequence alignment of FLN1 orthologues from the Ensembl Plants database indicates that FLN1 orthologues are found in most plants. The Gly-to-Arg substitution in the *e1493* mutant occurs in the highly conserved fpkB domain (Fig. S2). The FLN1 protein–protein interaction network is highly conserved in tomato, *Arabidopsis*, and rice (Fig. S4).

We used the virus-induced gene silencing (VIGS) technique to knock down the expression of *CaFLN1* in pepper. We also silenced *SlFLN1*, the orthologue of *CaFLN1* in tomato. Consistent with the albino phenotype of the *CaFLN1* mutant, both *CaFLN1*-silenced pepper plants and *SlFLN1*-silenced tomato plants developed chlorotic leaves (Fig. 1e, 1f). Using qRT-PCR, we demonstrated that the expression of *CaFLN1* and *SlFLN1* was significantly reduced in the VIGS plants (Fig. 1g). The chlorophyll content of the silenced plants was also significantly decreased, although the tomato VIGS plants accumulated normal levels of chlorophyll *a* (Fig. 1h). Our results demonstrated that *CaFLN1* contributes to the accumulation of chlorophyll in pepper and that the biological function of *FLN1* is conserved in pepper and tomato.

To gain mechanistic insight, we compared the transcriptomes of cotyledon tissue from MiniPep and *e1493*. The correlation coefficient among the three biological replicates reached 0.99–1 (Fig. S5a), and PC1 explained 95.8% of the total variance (Fig. S5b). A total of 5524 differentially expressed genes (DEGs) were identified, 2648 upregulated and 2876 downregulated (Fig. S5c). A Gene Ontology (GO) term enrichment analysis indicated that the missense mutation in *CaFLN1* led to changes in the expression of genes associated with many GO terms, including membrane and chloroplast development (Fig. S6a; Table S3). A KEGG pathway analysis indicated that *CaFLN1* is closely associated with photosynthesis (Fig. S6b; Table S4). An independent quantification of the relative expression of eight chloroplast-related DEGs using qRT-PCR validated the data from the RNA-seq experiment (Fig. S7a–b). We conclude that the missense mutation in the *CaFLN1* gene affects chloroplast development by disrupting PEP-mediated transcription.

In *Arabidopsis* and rice, FLN1 is an important subunit of the PEP complex and promotes chloroplast development [6, 8]. Multiple lines of evidence, such as high levels of sequence similarity; a highly conserved pfkB domain; phenotypic characterizations of mutants in rice, *Arabidopsis*, tomato, and pepper; a predicted protein–protein interaction network that is conserved; and our VIGS experiments are all consistent with FLN1 performing a conserved biological function in plants that is essential for chloroplast development. We speculate that the PEP complex is impaired in our pepper mutant because of a missense mutation in *CaFLN1* that consequently affects chloroplast biogenesis. Nevertheless, the exact mechanism remains to be elucidated.

## Supplementary Material

Web_Material_uhab084

## Data Availability

The RNA-seq and resequencing data from MiniPep and *e1493* are available at the NCBI repository (https://www.ncbi.nlm.nih.gov/bioproject/PRJNA770097).
