# Supplementary material for: Conserved role of fructokinase-like protein 1 in chloroplast development revealed by a seedling-lethal albino mutant of pepper
Source: Hortic Res. 2022 Jan 20;9:uhab084. doi: 10.1093/hr/uhab084 (PMC9016868; doi:10.1093/hr/uhab084)
Supplement: Web_Material_uhab084 [file web_material_uhab084.docx]

**Supplementary Document S1**

**This document includes the following contents:**

**Page 2-6: Supplementary Materials and Methods**

**Page 7-8: References for Supplementary Materials and Methods**

**Page 9-17: Supplementary Figures and Legends.**

**Page 9: Fig. S1** Mapped region (a), verified SNP variation (b), predicted subcellular location (c), and tissue expression (d) of *CaFLN1*.

**Page 10-11: Fig. S2** Multiple sequence alignment of FLN1 proteins in 18 Species.

**Page 12: Fig. S3** Phylogenetic tree of the pfkB family protein.

**Page 13: Fig. S4** Predicted protein interaction network for FLN1 in tomato, *Arabidopsis* and rice.

**Page 14: Fig. S5** RNA-seq analysis of MiniPep and *e1493* mutant.

**Page 15: Fig. S6** Gene ontology (a) and Kyoto Encyclopedia of Genes and Genomes pathway (b) enrichment analysis of the RNA-seq based differentially expressed genes (DEGs) between MiniPep and *e1493*.

**Page 16: Fig. S7** Expression of chloroplast related genes.

**Page 17: Fig. S8** Construction of VIGS vectors for *SlFLN1* and *CaFLN1*.

**Supplementary Materials and Methods**

**Plant materials and growth conditions**

The mutant *e1493* was derived from EMS mutagenesis of the miniature pepper cultivar, MiniPep (*Capsicum annuum* L.). The seeds were soaked in distilled water for 4 h and drained, transferred into 0.8% EMS solution and incubated in a shaker (50 r/min, 25℃) for 15 h. After the treatment, the EMS solution was recovered and the seeds were washed with tap water for 4 h. The seeds were germinated in an incubator at 28℃. Plants were grown in a growth chamber, with 25/20℃ day/night temperature, and 16/8 h light/dark cycle. The mutant *e1493* was identified in an M_2_ line (No. 1493), which showed albino and lethal phenotype at the cotyledonous stage. The phenotype was recorded by a digital camera when the mutant and wildtype (MiniPep) grew to 14 days of age. Genetic analysis of 42 M_2_ plants of the line showed 33 of them displaying wildtype phenotype while 9 seedlings displaying albino phenotype, suggesting the mutant was controlled by a recessive nuclear gene (χ^2^=0.127, *df* =1).

**Measurement of chlorophyll content**

Chlorophyll was extracted from the cotyledons of *e1493* mutant and MiniPep at 14 days of seedling age. The leaves of VIGS plants with clear phenotype were taken and ground with liquid nitrogen, and 0.1 g powder was used for extraction. Three biological replicates were prepared for each genotype. Chlorophyll was extracted with 1 mL 95% ethanol at 4℃ for 12 h under darkness and then centrifuged at 15000 rpm for 10 min. Using 95% ethanol as blank, the absorbance value of chlorophyll extract was measured at 646 nm and 663 nm using a microplate reader (BMG Labtech, Germany) and the calculation of chlorophyll content was based on a published paper^1^.

**Observation of chloroplast structure**

The chloroplast structure in the cotyledons of *e1493* and the wildtype was checked by transmission electron microscope (TEM). The cotyledons of 14 days old seedling were taken, cut into 0.3 cm×0.3 cm pieces, and quickly fixed in 2.5% glutaraldehyde solution. Vacuum was used to help the samples to settle to the bottom, and the fixation was performed at 4℃ for no less than 4 h. The fixed samples were washed with 0.1 M phosphate buffer (pH7.0), followed by fixing in 1% osmiophilic tetroxide before dehydration using gradient alcohol solution. The samples were embedded in Spurr resin for ultra-thin slicing. Images were captured using a TEM (Hitachi-7650, Japan). Details about sample preparation and observation were based on a published reference^2^. The sample preparation was commissioned by the Core Facility and Technical Support, Wuhan Institute of Virology, Chinese Academy of Sciences.

**Bulked segregant RNA-Seq (BSR)**

To clone the causative gene underlying *e1493* mutant, an F_2_ population was constructed using a heterozygous mutant plant as the female and pepper cultivar PC69 (*C. annuum*) as the male parent. A total of 500 F_2_ plants were grown, the cotyledons of 30 seedlings displaying the mutant or wildtype phenotype, were collected in respective from 14-d-old seedlings to construct two pools. Total RNA was extracted from each pool with TRIzol reagent (Invitrogen, Carlsbad, CA, USA) according to the product instruction. Qualified RNA samples were sent to Novogen Ltd. (Tianjin, China) for library construction and sequencing with HiSeq4000 platform (Illumina, USA). Raw reads were filtered using Fastp^3^ to remove reads with adaptors, low quality and high content of unknown bases. Clean reads were mapped to the pepper reference genome (CM334 V1.6)^4,5^ using Hisat2 (ref.6). The sam files obtained from mapping were converted into compressed and sorted bam files using Samtools view and Samtools sort^7^. Then, SNPs were identified and extracted using Samtools mpileup^7^ and Bcftools^8^. The SNP-index of each pool was calculated, the reads depth for each SNP less than 10 in either pool or SNP-index less than 0.3 or greater than 0.7 in both pools were filtered out to reduce the interference of alignment errors. Then, the ∆SNP-index between the two pools, and the Euclidian distance (ED value) of each SNP was calculated. The ED value was further processed to the power of 4 (ED^4^), and then linear regression fitting is carried out for the data using the Loess curve^9^. The 3 times of standard deviations of ED^4^ power fitting values plus median value of all SNPs was taken as the threshold line, and the region higher than the threshold line was taken as the candidate region.

**Identification of the candidate gene**

To identification of variation between the mutant and wildtype in the mapped region, *e1493* and MiniPep were resequenced. DNA was extracted from cotyledons by classical CTAB method and sent to Novogen Ltd. for library construction and sequencing with Hiseq4000 (150 bp paired-end sequencing, PE150). Raw reads were filtered by Fastp^3^, and clean reads were mapped to the pepper reference genome (CM334 V1.6)^4,5^ using bwa mem^10^. Duplicated reads derived from PCR were marked with Picard (<http://broadinstitute.github.io/picard/>). The generated bam file was used to identify SNPs and InDels using GATK^11^. SNPs were filtered using bcftools^8^ with the parameters “QD < 2.0 || FS > 60.0 || MQ< 40.0 || MQRankSum < -12.5 || ReadPosRankSum < -8.0”. The retained SNPs were further annotated using SnpEff^12^. To identify causative mutations, priority was given to SNPs meeting the following criteria: (1) Homologous SNPs unique to *e1493*; (2) SNPs from G/C to A/T substitution; (3) SNPs located in coding region that causes amino acid change, such as missense mutation. The finally retained SNPs were amplified from both MiniPep and *e1493* using Phanta Max super-fidelity DNA Polymerase (Vazyme, Nanjing, China) with specific primers (Table S5) and verified by Sanger sequencing.

In addition, the tissue expression profile of the causative gene, *CaFLN1*, was retrieved from our released PepperHub database^13^. And the prediction of the subcellular localization of CaFLN1 was performed online (<https://www.uniprot.org/>). The protein-protein interaction networks for FLN1 in tomato, *Arabidopsis* and rice were predicted using the STRING database (https://string-db.org), which has not yet included pepper species. Cytoscape software^14^ was used to visualize the network.

**Phylogenetic tree and conservation analysis of FLN1**

The Hidden Markov Model (HMM) of the fpkB domain was retrieved from Pfam database (<https://pfam.xfam.org>). HMMER hmmscan program (<https://www.ebi.ac.uk/Tools/hmmer/>, V3.0) was used to identify fpkB gene family members. InterProScan^15^ and Pfam (<https://www.genome.jp/tools/motif/>) were further used to confirm that all the members include fpkB domain. Amino acid sequence (Table S6) alignment was performed using ClustalW program in MEGA X^16^, with bootstrap value of 1000 and the best model to construct the maximum likelihood (ML) tree. The tree was finally displayed with iTOL^17^ (<https://itol.embl.de/>). The amino acid sequences of FLN1 homologs were retrieved from the Ensemble Plant database (<https://plants.ensembl.org/index.html>), and the sequences from 18 species (Table S7) were selected for alignment. The multiple sequence alignment for FLN1 was performed by mafft^18^ and visualized by Jalview^19^.

**Virus induced gene silencing (VIGS) of FLN1**

VIGS was performed essentially according to previous reports^20^. For construction of VIGS vectors, Primer 3 (<https://primer3.ut.ee/>) was used to design the primers (Table S5). A fragment of 317 bp and 332 bp were amplified in respective from *CaFLN1* and *SlFLN1*, and then recombined into *EcoRI*-linearized pTRV2 vectors (designed pTRV2- pTRV2-*CaFLN1* and pTRV2-*SlFLN1*, respectively) (Fig. S8). Meanwhile, a fragment of *PDS* gene from tomato and pepper was also recombined into *EcoRI*-linearized pTRV2 vectors, which served as the positive control. Fig. S5b shows detailed information about pTRV2 vectors. The method of homologous recombination referred to the instructions of the ClonExpress II One Step Cloning Kit (Vazyme, Nanjing, C112-01). The vectors pTRV1, pTRV2, pTRV2-*CaPDS*, pTRV2-*SlPDS*, pTRV2-*CaFLN1* and pTRV2-*SlFLN1* were introduced into *Agrobacterium tumefaciens* (GV3101) *via* electroporation. Agrobacterial suspensions carrying the vectors mentioned above were adjusted to OD_600_=0.5, and the suspensions with pTRV1 were mixed equally with the suspensions of pTRV2 or pTRV2 derived vectors. Seedlings of the wildtype pepper MiniPep at 12 days of seedling age and tomato cultivar Alisa Craig (*Solanum lycopersicum*) at 8 days of seedling age were used for inoculation. Agrobacterial suspensions were infiltrated into the underside of the cotyledons. After inoculation, the plants were cultured in dark for 3 d and then transferred to the growth chamber. The changes of plant phenotype were observed and photographed at the seedling age of 30 days.

**Transcriptome analysis**

Cotyledons from six plants of either the wildtype (MiniPep) or the mutant (*e1493*) at 14 days of seedling age were collected as one biological replicate, and three biological replicates were prepared for each genotype. RNA extraction, library construction, raw reads filtering, and clean reads mapping and sorting were essentially the same as BSR method. After generation of the bam files, featureCounts^21^ was employed to calculate the raw counts for each gene. To eliminate the effects of gene size and sequencing depth, the fragments per kb per million fragments (FPKM) method^22^ was used to estimate gene expression levels. To ensure the accuracy of subsequent analysis, two methods were used to evaluate the repeatability of biological replicates both based on FPKM value. Principal component analysis (PCA) was used for clustering analysis of the replicates. Meanwhile, the function cor built-in R program was used to calculate the Pearson correlation coefficient between two samples, and then the R package Corrplot was used to plot the heatmap of correlation coefficients. After obtaining the quantitative gene expression matrix (raw counts matrix), DEseq2 (ref.23) was used to identify differentially expressed genes (DEGs) between the wildtype and the mutant. DEGs were defined as |log_2_ (Fold Change)|≥1 and with false discovery rate less than 0.05 (FDR<0.05)^24^. For Gene Ontology (GO) enrichment analysis, all the DEGs were mapped to the GO database (http://geneontology.org/), and the number of transcripts for each GO term was calculated. Then the hypergeometric test was applied to find out the significantly enriched GO terms, with q-value ≤ 0.05 as the criterion. Annotation and enrichment analysis of Kyoto Encyclopedia of Genes and Genomes (KEGG) pathway was performed with KOBAS3.0 (ref.25) with p-value ≤ 0.05 as the criterion.

**Quantitative reverse-transcribed PCR (qRT-PCR) analysis**

The backup samples for the transcriptome analysis above were used for validation. The samples from VIGS experiments were taken from 30 days-old VIGS plants. All the samples consisted of three biological replicates. Total RNA was extracted using TRIzol reagent, and one microgram of total RNA was reverse transcribed using HiScript II 1st Strand cDNA Synthesis Kit (Vazyme) according to the supplier’s instruction. SYBR Green-based qRT-PCR was performed on a QuantStudio 7 platform (Thermo Fisher Scientific), with a 10 μL reaction system. Pepper *UBI-3* (ref.26) and tomato *SlFRG37* (ref.27) were used as internal control, respectively. All the primer sequences used for qRT-PCR were listed in Table S5. The cycling conditions were as follows: pre-denaturation at 95℃ for 1 min; 95℃ 10 s; 58℃ 15 s, 72℃ 20 s, 40 cycles, followed by a standard melting curve analysis. The data were processed with the 2^-ΔΔCT^ method^28^.

**References for Supplementary Materials and Methods**

1. Wang, K., Hersh, H.L. & Benning, C. Sensitive to freezing2 aides in resilience to salt and drought in freezing-sensitive tomato. *Plant Physiol.* **172**, 1432-1442 (2016).

2. Fan, M. et al. Overexpression of *SlRBZ* results in chlorosis and dwarfism through Impairing chlorophyll, carotenoid, and gibberellin biosynthesis in tomato. *Front. Plant Sci.* **7**, 907 (2016).

3. Chen, S., Zhou, Y., Chen, Y. & Gu, J. Fastp: an ultra-fast all-in-one Fastq preprocessor. *Bioinformatics* **34**, i884-i890 (2018).

4. Kim, S. et al. Genome sequence of the hot pepper provides insights into the evolution of pungency in *Capsicum* species. *Nat. Genet.* **46**, 270-278 (2014).

5. Kim, S. et al. New reference genome sequences of hot pepper reveal the massive evolution of plant disease-resistance genes by retroduplication. *Genome Biol.* **18**, 210 (2017).

6. Kim, D., Paggi, J.M., Park, C., Bennett, C. & Salzberg, S.L. Graph-based genome alignment and genotyping with Hisat2 and Hisat-genotype. *Nat. Biotechnol.* **37**, 907-915 (2019).

7. Li, H. et al. The sequence alignment/map format and SAMtools. *Bioinformatics* **25**, 2078-2079 (2009).

8. Danecek, P. & McCarthy, S.A. BCFtools/csq: haplotype-aware variant consequences. *Bioinformatics* **33**, 2037-2039 (2017).

9. Hill, J.T. et al. MMAPPR: mutation mapping analysis pipeline for pooled RNA-seq. *Genome Res.* **23**, 687-697 (2013).

10. Li, H. & Durbin, R. Fast and accurate short read alignment with Burrows-Wheeler transform. *Bioinformatics* **25**, 1754-1760 (2009).

11. McKenna, A. et al. The genome analysis toolkit: a mapReduce framework for analyzing next-generation DNA sequencing data. *Genome Res.* **20**, 1297-1303 (2010).

12. Cingolani, P. et al. A program for annotating and predicting the effects of single nucleotide polymorphisms, SnpEff: SNPs in the genome of *Drosophila melanogaster* strain w1118; iso-2; iso-3. *Fly (Austin)* **6**, 80-92 (2012).

13. Liu, F. et al. PepperHub, an Informatics Hub for the Chili Pepper Research Community. *Mol. Plant* **10**, 1129-1132 (2017).

14. Otasek, D., Morris, J.H., Bouças, J., Pico, A.R. & Demchak, B. Cytoscape automation: empowering workflow-based network analysis. *Genome Biology* **20**, 185 (2019).

15. Jones, P. et al. InterProScan 5: genome-scale protein function classification. *Bioinformatics* **30**, 1236-1240 (2014).

16. Kumar, S., Stecher, G., Li, M., Knyaz, C. & Tamura, K. MEGA X: molecular evolutionary genetics analysis across computing platforms. *Mol. Biol. Evol.* **35**, 1547-1549 (2018).

17. Letunic, I. & Bork, P. Interactive tree of life (iTOL) v3: an online tool for the display and annotation of phylogenetic and other trees. *Nucleic Acids Res.* **44**, W242-W245 (2016).

18. Katoh, K., Misawa, K., Kuma, K.i. & Miyata, T. MAFFT: a novel method for rapid multiple sequence alignment based on fast fourier transform. *Nucleic Acids Res.* **30**, 3059-3066 (2002).

19. Clamp, M., Cuff, J., Searle, S.M. & Barton, G.J. The Jalview Java alignment editor. *Bioinformatics* **20**, 426-427 (2004).

20. Wang, J.E., Li, D.W., Gong, Z.H. & Zhang, Y.L. Optimization of virus-induced gene silencing in pepper (*Capsicum annuum* L*.*). *Genet. Mol. Res.* **12**, 2492-2506 (2013).

21. Liao, Y., Smyth, G.K. & Shi, W. FeatureCounts: an efficient general purpose program for assigning sequence reads to genomic features. *Bioinformatics* **30**, 923-930 (2014).

22. Conesa, A. et al. A survey of best practices for RNA-seq data analysis. *Genome Biology* **17**, 13 (2016).

23. Wang, L., Feng, Z., Wang, X., Wang, X. & Zhang, X. DEGseq: an R package for identifying differentially expressed genes from RNA-seq data. *Bioinformatics* **26**, 136-138 (2010).

24. Love, M.I., Huber, W. & Anders, S. Moderated estimation of fold change and dispersion for RNA-seq data with DESeq2. *Genome Biology* **15**, 550 (2014).

25. Bu, D. *et al.* KOBAS-i: intelligent prioritization and exploratory visualization of biological functions for gene enrichment analysis. *Nucleic Acids Research* **49**, W317-W325 (2021).

26. Wan, H. et al. Identification of reference genes for reverse transcription quantitative real-time PCR normalization in pepper (*Capsicum annuum* L.). *Biochem. Biophys. Res. Commun.* **416**, 24-30 (2011).

27. Cheng, Y. et al. Genome-wide identification and evaluation of reference genes for quantitative RT-PCR analysis during tomato fruit development. *Front. Plant Sci.* **8**, 1440 (2017).

28. Schmittgen, T.D. & Livak, K.J. Analyzing real-time PCR data by the comparative C(T) method. *Nat. Protoc.* **3**, 1101-1108 (2008).

**Supplementary Figures and Legends**

**
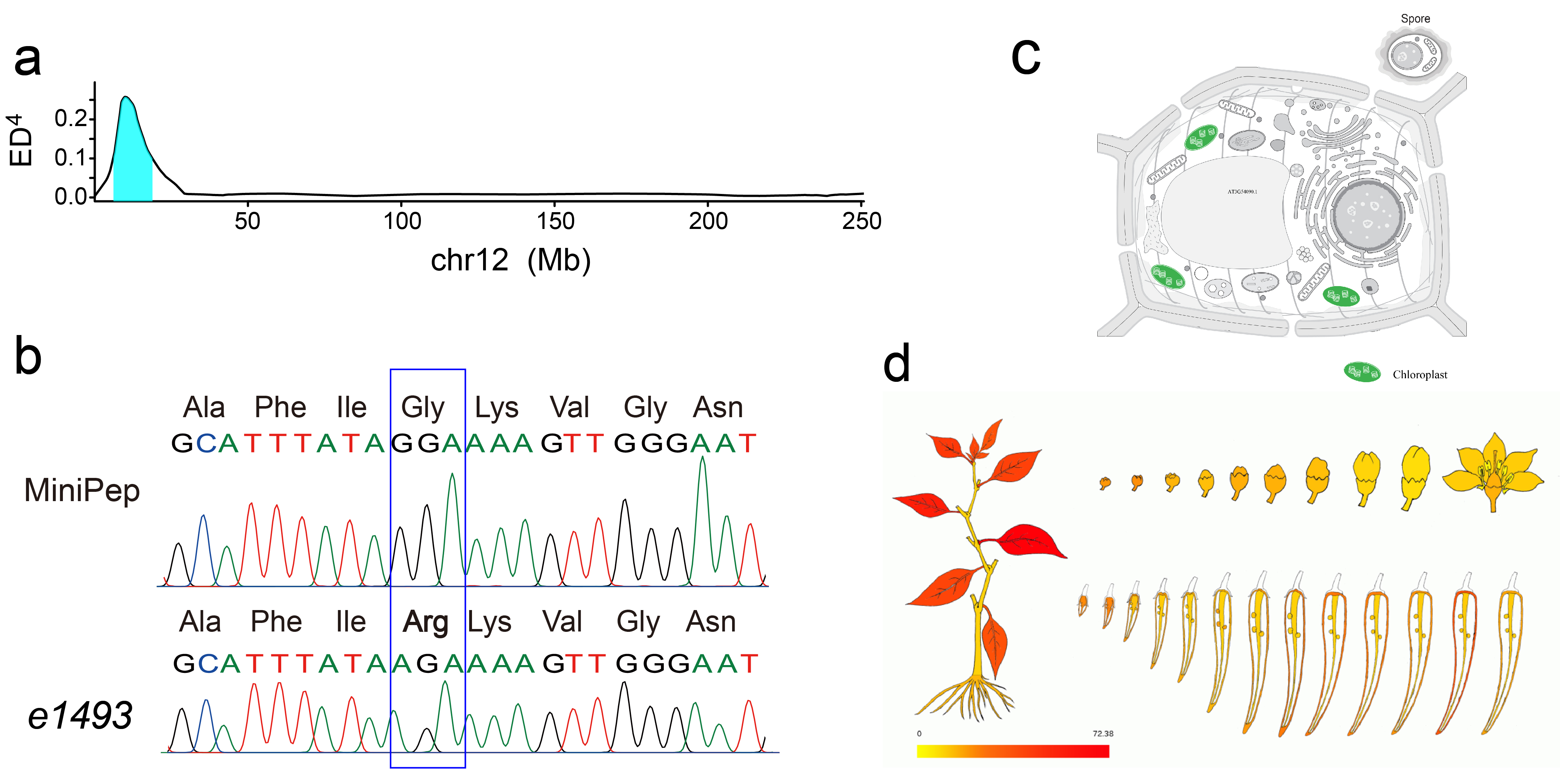
**

**Fig. S1 Mapped region (a),** [**verified**](javascript:;) **SNP variation (b), predicted subcellular location (c), and tissue expression (d) of *CaFLN1*.** CaFLN1 was mapped on chromosome 12 at the position of 6.1 M to 18.9 M using bulked segregant RNA-Seq (BSR), and the causative single-nucleotide polymorphism (SNP, chr12_12913738) was verified using Sanger sequencing and the corresponding change of amino acid from glycine (Gly) to arginine (Arg) is shown in the blue box. The subcellular localization of CaFLN1 was predicted online (<https://www.uniprot.org/>), and the tissue expression cartoon was derived from PepperHub database (PepperHub, http://www.hnivr.org/pepperhub/).

**
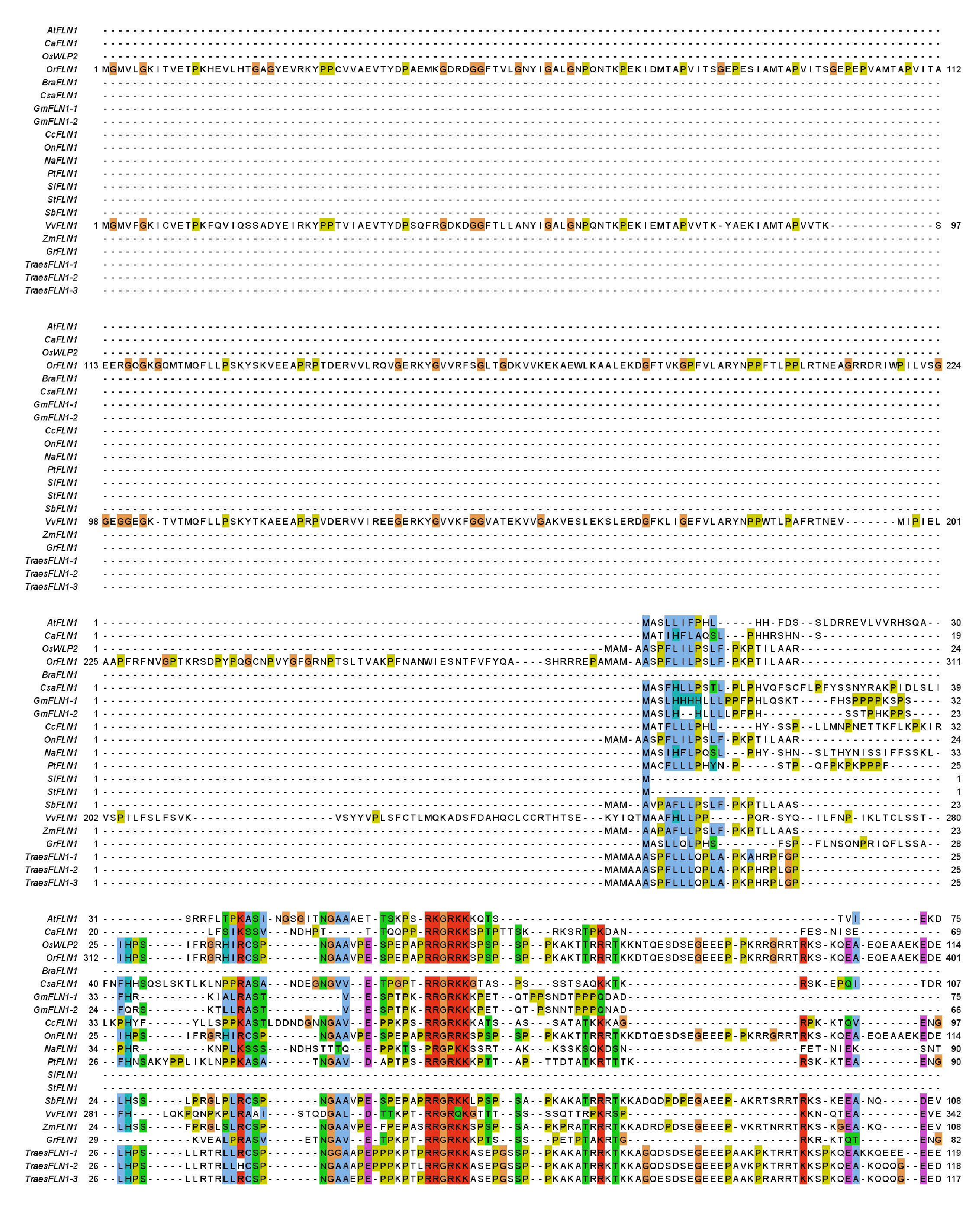
**

Continue with the next page

**
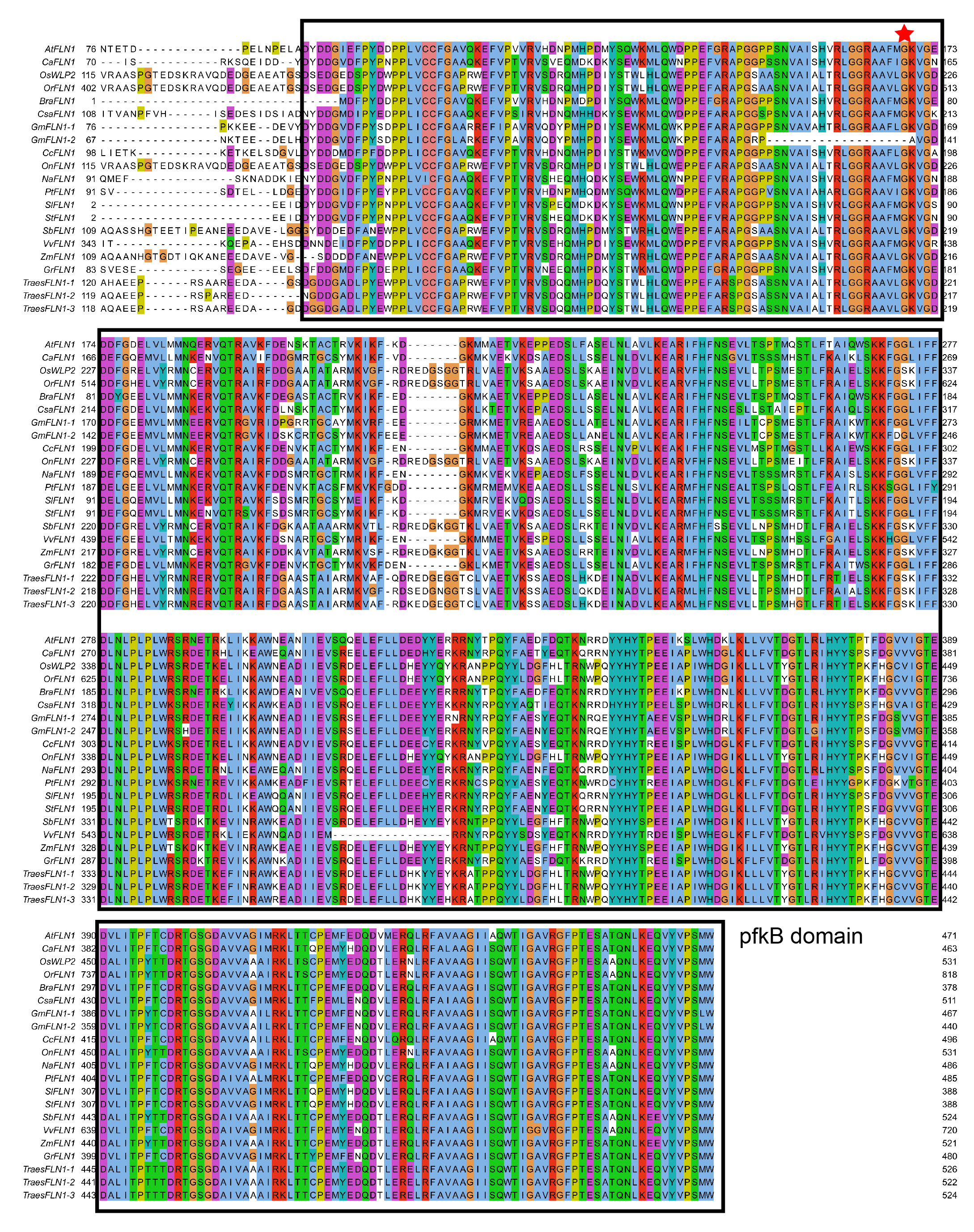
**

**Fig. S2 Multiple sequence alignment of FLN1 proteins in 18 Species.** The alignment was conducted using ClustalW program in MEGA X. The colours correspond to amino acid identity, and the conserved pkfB domain is indicated by the black box.

**
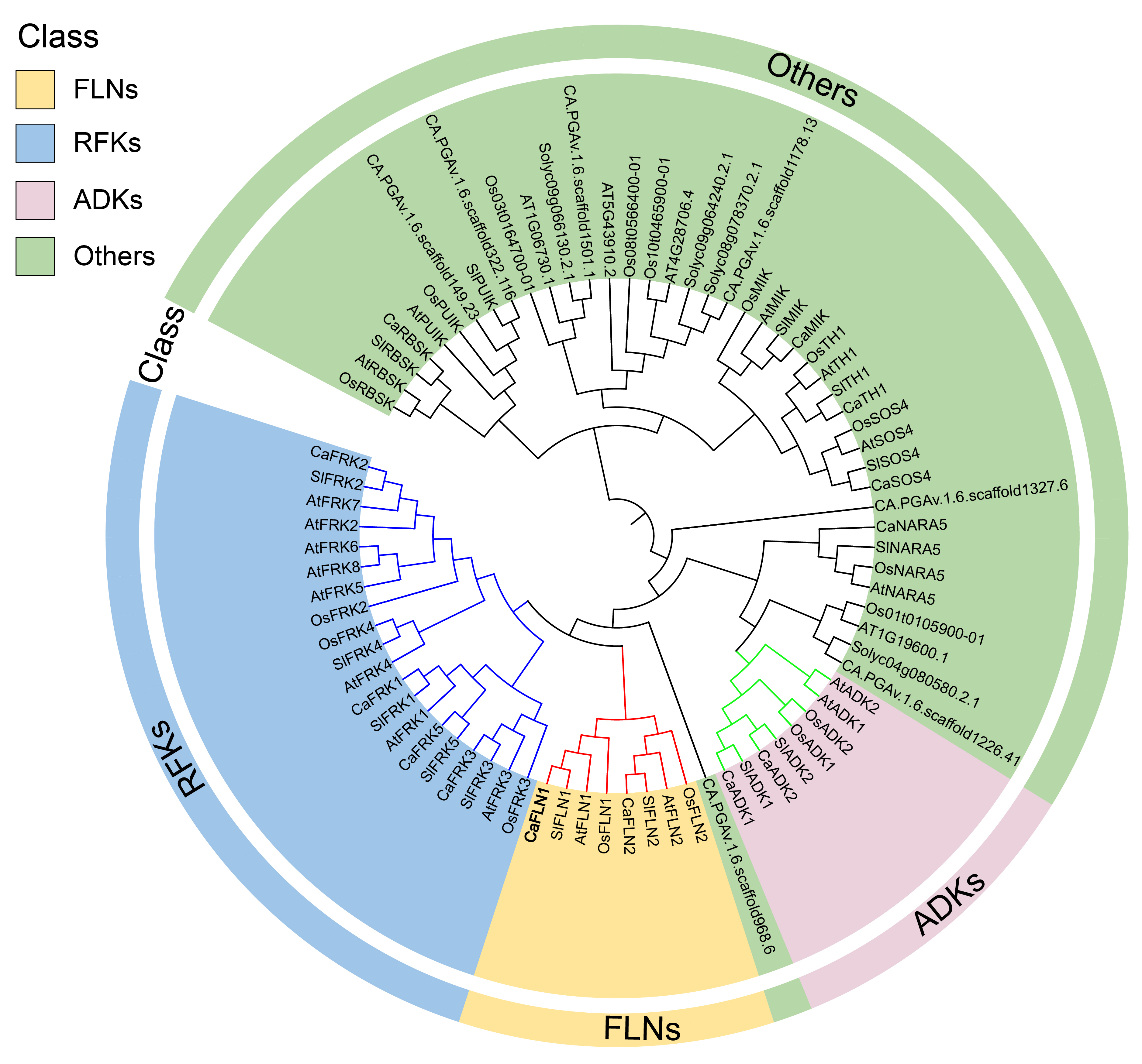
**

**Fig. S3 Phylogenetic tree of the pfkB family protein.** A maximum likelihood tree was constructed for the 78 pfkB members from pepper, tomato, *Arabidopsis* and rice, using MEGA X with bootstrap value of 1000. CaFLN1 was marked in blod. Different-colored arcs indicate different subfamilies.

**
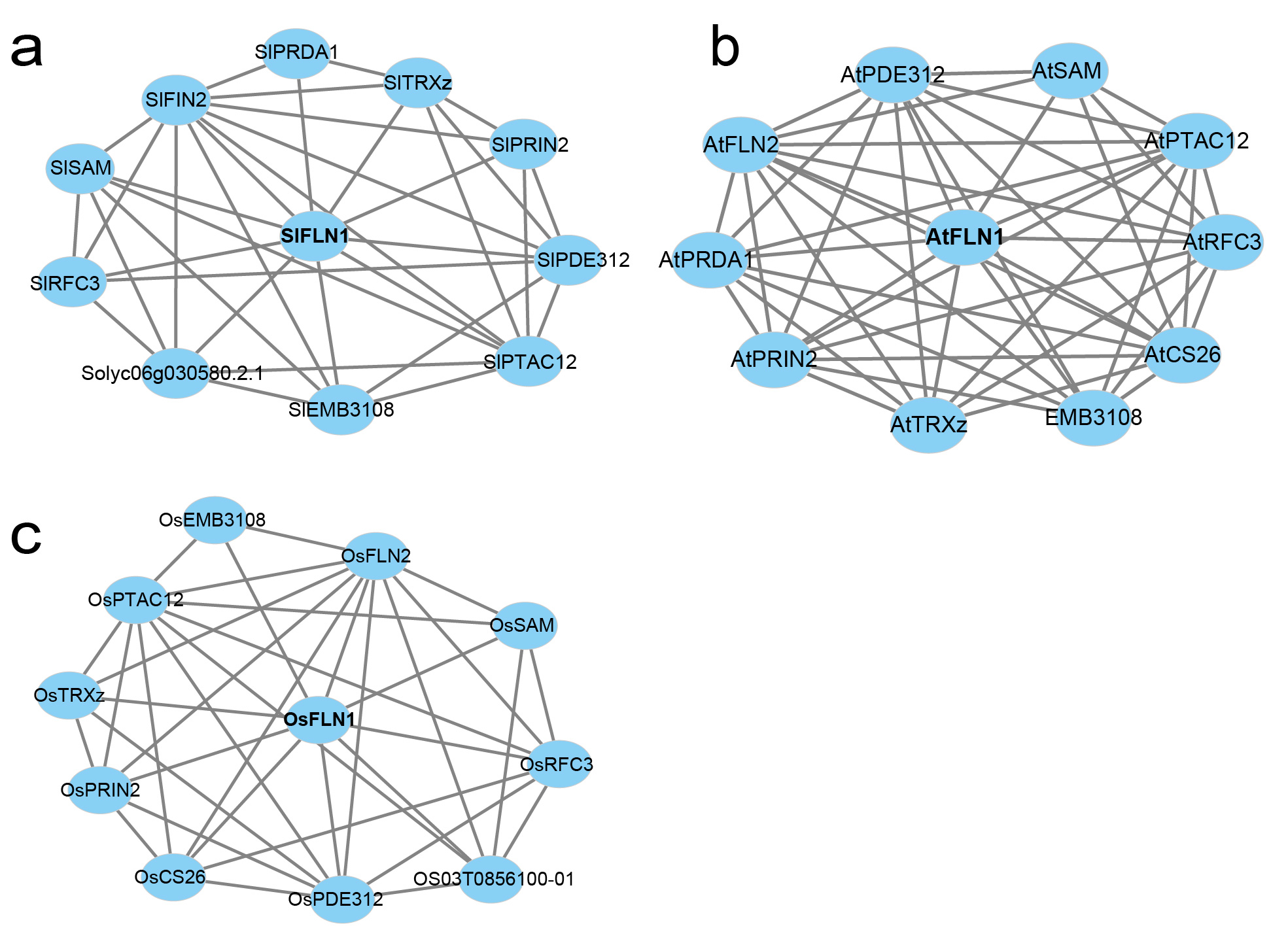
**

**Fig. S4 Predicted protein interaction network for FLN1 in tomato, *Arabidopsis* and rice.** The prediction was performed using STRING database (https://string-db.org/).

**
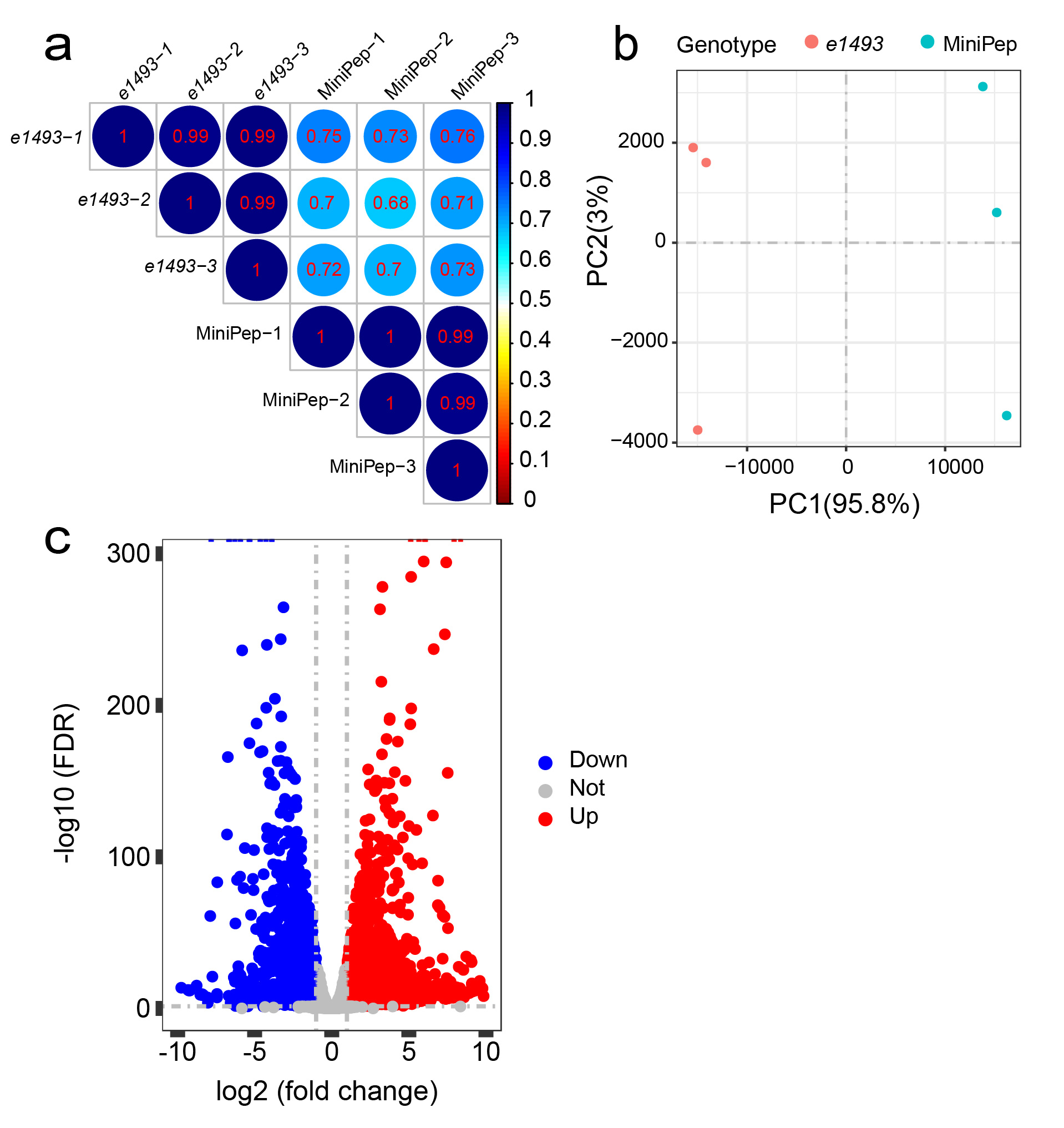
**

**Fig. S5 RNA-seq analysis of MiniPep and *e1493* mutant.** (a) Correlation analysis among samples. Spearman represents: 0.8-1.0 very strong correlation; 0.6-0.8 strong correlation. (b) PCA analysis. X axis represents PC1 level, and Y axis represents PC2 level. (c) Volcano map of differentially expressed genes (DEGs) based on RNA-seq. The red, blue and gray dots represent upregulated DEGs (Up), downregulated DEGs (Down), and not change genes (Not), respectively.

**
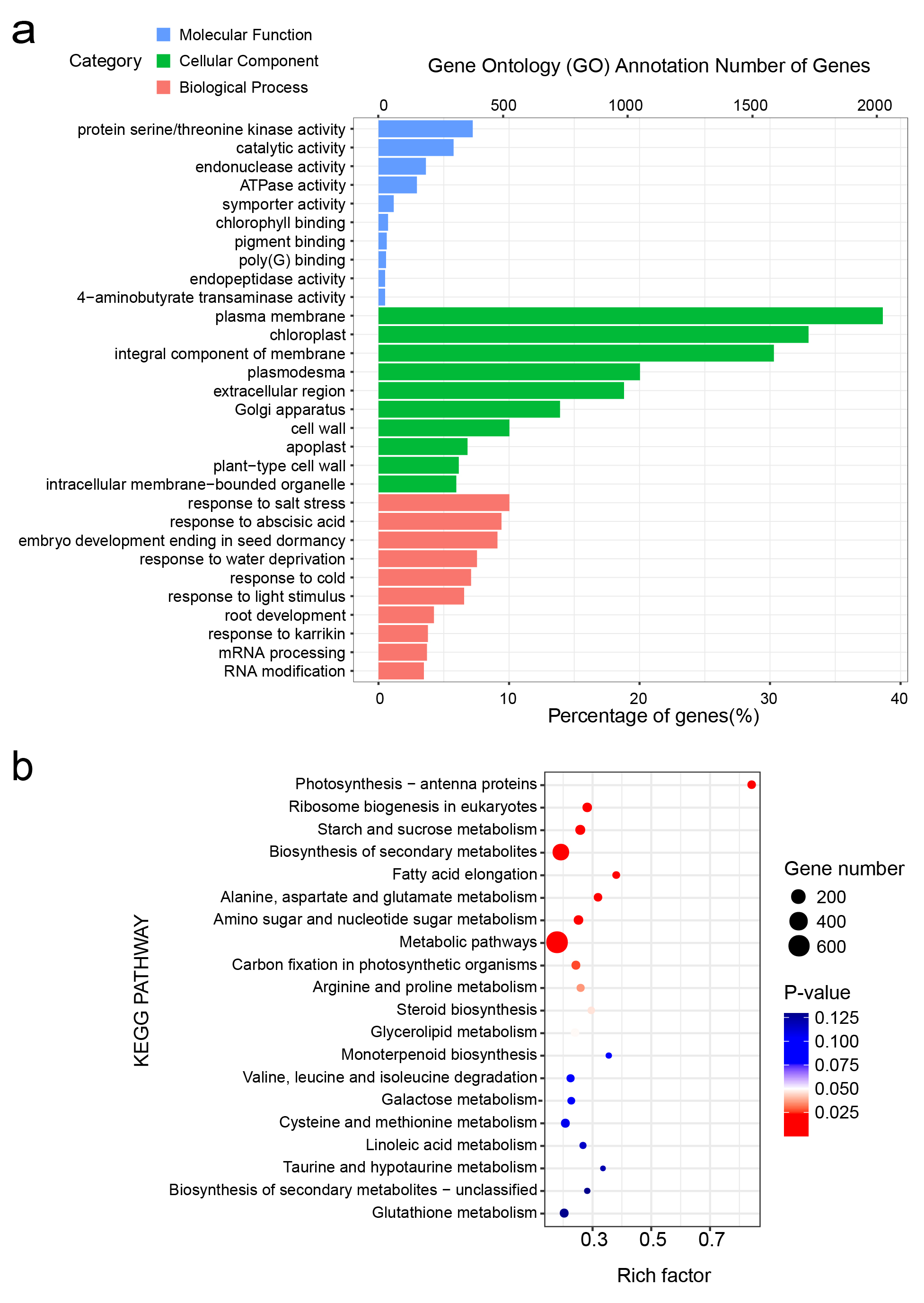
**

**Fig. S6 Gene ontology (a) and Kyoto Encyclopedia of Genes and Genomes pathway (b) enrichment analysis of the RNA-seq based differentially expressed genes (DEGs) between MiniPep and *e1493*.** RNA-seq was performed on the cotyledonous tissue of the wildtype (MiniPep) and *e1493* mutants at 14 days of seedling age. In (a), Y axis represents the GO classification, the bottom and top of the X axis represents the percentage and the number of genes, respectively. In (b), Y axis represents the pathway name, and X axis represents the enrichment factor. The larger the enrichment factor, the higher enrichment level of DGEs in the pathway. P-value is expressed by color with white as the limit. The deeper the red color, the more reliable the significance of the enrichment of DEGs in the pathway. The number of DEGs in a pathway was expressed by the size of the circle. The larger the circle, the higher enrichment of this pathway.

**
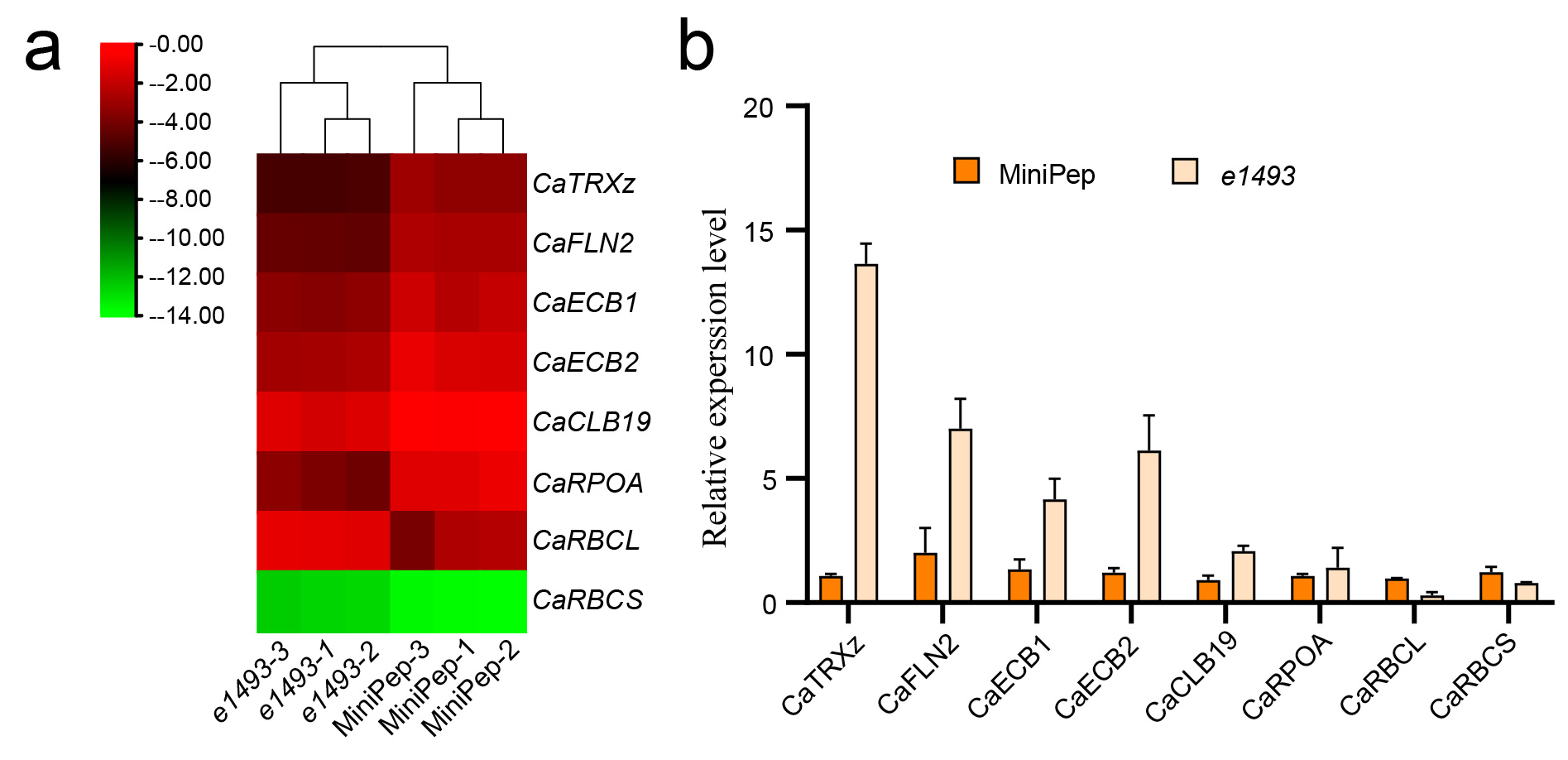
**

**Fig. S7 Expression of chloroplast related genes.** (a) Heatmap showing the expression of chloroplast related genes based on RNA-seq. (b) Relative expression of chloroplast related genes based on quantitative reverse-transcribed PCR (qRT-PCR). *CaRBCL* and *CaRBCS* are PEP-dependent plastid genes. Error bars indicate standard deviation (n = 3)

**
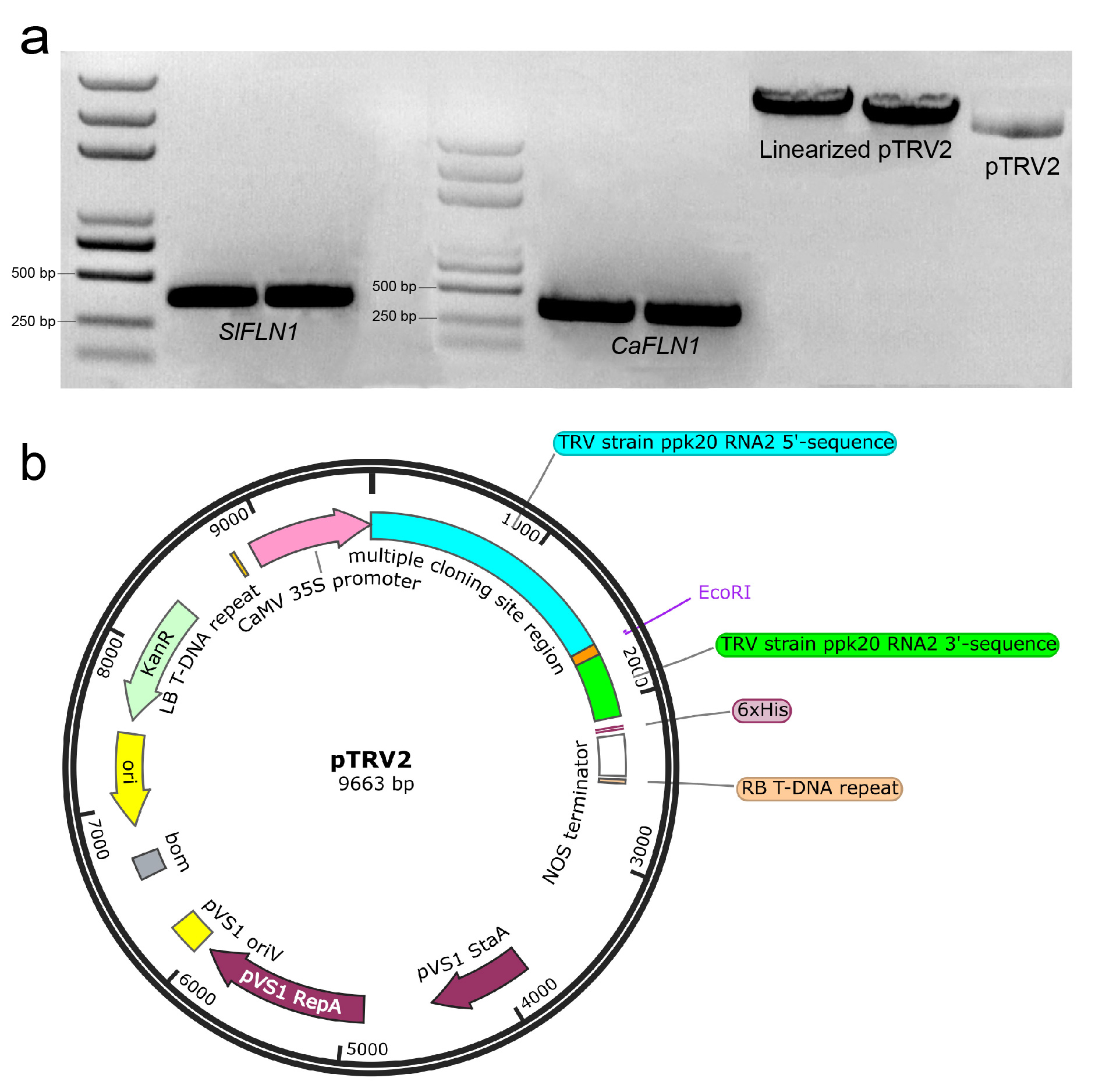
**

**Fig. S8 Construction of VIGS vectors for *SlFLN1* and *CaFLN1*.** (a) The amplification of a fragment from *SlFLN1* and *CaFLN1*, and digestion of pTRV2 vector with *EcoR*I enzyme. (b) Vector map of pTRV2.
